# Dairy Lactic Acid Bacteria and Their Potential Function in Dietetics: The Food–Gut-Health Axis

**DOI:** 10.3390/foods10123099

**Published:** 2021-12-14

**Authors:** Duygu Ağagündüz, Birsen Yılmaz, Teslime Özge Şahin, Bartu Eren Güneşliol, Şerife Ayten, Pasquale Russo, Giuseppe Spano, João Miguel Rocha, Elena Bartkiene, Fatih Özogul

**Affiliations:** 1 Department of Nutrition and Dietetics, Gazi University, Emek, Ankara 06490, Turkey; dytbirsen@gmail.com (B.Y.); tozgeyrsn@gmail.com (T.Ö.Ş.); dyt.beg@gmail.com (B.E.G.); serife_9507@hotmail.com (Ş.A.); 2 Department of Nutrition and Dietetics, Cukurova University, Sarıcam, Adana 01380, Turkey; 3 Department of Agriculture, Food, Natural Science, Engineering, University of Foggia, Via Napoli 25, 71122 Foggia, Italy; pasquale.russo@unifg.it (P.R.); giuseppe.spano@unifg.it (G.S.); 4 Laboratory for Process Engineering, Environment, Biotechnology and Energy (LEPABE), Department of Chemical Engineering (DEQ), Faculty of Engineering, University of Porto FEUP, 4200-465 Porto, Portugal; jmfrocha@fe.up.pt; 5 Department of Food Safety and Quality, Institute of Animal Rearing Technologies, Lithuanian University of Health Sciences, Tilzes 18, LT-47181 Kaunas, Lithuania; elena.bartkiene@lsmuni.lt; 6 Department of Seafood Processing Technology, Faculty of Fisheries, Cukurova University, Balcali, Adana 01330, Turkey; fozogul@cu.edu.tr

**Keywords:** lactic acid bacteria, dairy food products, gut microbiota, health benefits, disease prevention

## Abstract

Fermented dairy products are the good source of different species of live lactic acid bacteria (LAB), which are beneficial microbes well characterized for their health-promoting potential. Traditionally, dietary intake of fermented dairy foods has been related to different health-promoting benefits including antimicrobial activity and modulation of the immune system, among others. In recent years, emerging evidence suggests a contribution of dairy LAB in the prophylaxis and therapy of non-communicable diseases. Live bacterial cells or their metabolites can directly impact physiological responses and/or act as signalling molecules mediating more complex communications. This review provides up-to-date knowledge on the interactions between LAB isolated from dairy products (dairy LAB) and human health by discussing the concept of the food–gut-health axis. In particular, some bioactivities and probiotic potentials of dairy LAB have been provided on their involvement in the gut–brain axis and non-communicable diseases mainly focusing on their potential in the treatment of obesity, cardiovascular diseases, diabetes mellitus, inflammatory bowel diseases, and cancer.

## 1. Introduction

Fermented milk and dairy products are the milestones of dietary lifestyle around the world. Beyond their nutritional and organoleptic properties, the health benefits of fermented dairy products have long been known [1]. In particular, fermented dairy products are a good source of different species of live lactic acid bacteria (LAB), and beneficial microbes are well characterized for their probiotic potential [2]. Probiotics are live microorganisms which, when administered in adequate amounts, confer a health benefit on the host [3]. The ingestion of probiotics harmonises the composition of commensal microorganisms of the intestinal and urogenital environment by competing with pathogens for nutrients and binding sites and by the in situ production of antimicrobial metabolites [4]. In addition, probiotics contribute to improving the mucosal barrier function by modulating immune responses in the host and controlling symptoms of inflamed gastrointestinal conditions like those observed in inflammatory bowel disease (IBD) [5]. Less obvious is the contribution that dairy LAB may exert in the prevention and therapeutic of different human diseases including gynaecological, reproductive, metabolic, cardiovascular, osteoporosis, as well as apoptosis control [6]. 

The complex enzymatic heritage of LAB strains contributes to the release of several bioactive metabolites either into the dairy matrix or in the gut, thus promoting the fascinating concept of the food–gut-health axis. These metabolites directly affect different physiological processes or act as signalling molecules to surrounding microorganisms. For example, encrypted bioactive peptides mainly produced by dairy LAB from the catabolism of alpha and beta-caseins, albumin, and globulins have been reported as anti-microbials, hypercholesteraemics, opioid and opioid antagonists, angiotensin-converting enzyme inhibitors, anti-thrombotics, immunomodulators, cytomodulators, and anti-oxidants [7]. There is evidence that the consumption of probiotics-containing dairy products such as yogurt, cultured fermented milk, and kefir has been associated with a range of health benefits including cholesterol metabolism and angiotensin-converting enzyme (ACE) inhibition, antimicrobial activity, tumour suppression, increased speed of wound healing, and the modulation of the immune system [8,9]. Recently, dairy products fermented by Lactobacillus strains were reported to modulate the gut-bone axis in a murine model; this is an effect that can be modulated by living Lactobacillus cells as well as dairy products fermented by the same *Lactobacillu*s [10]. In the food–gut complex ecosystem, dairy LAB can induce a network of signals mediated by the whole bacteria or their components. The gut–brain–microbiota axis is based on a bilateral communication system through signalling from gut-microbiota to brain and from brain to gut-microbiota by the involvement of neural, endocrine, immune, and metabolic links [11]. In a preliminary study, Butler and co-authors observed that dietary changes based on the intake of dairy products promote the *Lactobacillus* abundance and suggest a link with the psychological status in participants by measuring the predictive neuroactive potential using a gut–brain module approach [12]. Accordingly, a recent survey indicated that the intake of dairy products such as milk, yogurt, and kefir may modulate the gut microbiota by increasing the *Lactobacillus* population [13], while consumption of a fermented dairy product containing a probiotic *Lactobacillus casei* strain reduces the duration of respiratory infections in the elderly [14]. However, the influence of dairy consumption in conjunction with other factors such as host genetics, age, sex, and lifestyle influences the gut microbiota composition and functionality. 

This review generally highlights the complex interactions between LAB isolated from dairy products (i.e., dairy LABs) and human health proposing the novel concept of the food-gut-health axis. In particular, the article focuses on some fermentation and/or probiotic potentials of dairy LAB and their involvement in the gut–brain axis as well as of non-communicable diseases (NDCs) and mostly on their protective potential in the treatment of obesity, cardiovascular diseases, diabetes mellitus, inflammatory bowel diseases, and cancer.

## 2. Definitions and Some Characteristics of LABs 

LAB are a phylogenetically and functionally diverse group of bacteria [15] and are widely known as microbial food cultures [16]. By definition, LAB constitute a phylogenetically and functionally diverse taxonomic order of bacteria [15]. These diverse microorganisms have a common defining characteristic: they produce lactic acid as the end result in the process of fermenting carbohydrates [17,18,19]. Traditionally, they are closely linked to the fermentation of human foods especially in dairy products [16]. The general characteristics seen in LAB are summarized in Table 1. 

## 3. Importance of LABs in Dairy Foods in Terms of Health 

LAB play a key role in the positive health effects of fermented milks and dairy products. LAB can sometimes be found naturally in some dairy products, or they can be added as a starter culture or sometimes novel ingredients/additives for increasing the functionality especially for probiotic potential of the product even if there are some unclear matters. LAB are a group of bacteria often found in fermented dairy foods that include genera such as *Lactobacillus*, *Lactococcus*, *Pediococcus*, *Enterococcus*, and *Streptococcus* [21]. Most bacteria used as probiotics belong to the LAB species, genus *Lactobacillus* (*Lactobacillus acidophilus*, *Limosilactobacillus fermentum*, *Lacticaseibacillus casei*, *Limosilactobacillus reuteri*, *Lactocaseibacillus rhamnosus*, *Lactobacillus helveticus*, *Lactococcus lactis*, *Lactobacillus crispatus*, *Lactobacillus gasseri*, and *Lactiplantibacillus plantarum*) and genus *Enterococcus* (*Enterococcus faecalis* and *Enterococcus faecium*) [22]. Since LAB starter cultures have been used for many years to carry out food and milk fermentation, these bacteria are generally considered ‘generally recognized as safe’(GRAS) [23].

The main factors affecting the nutritional value of dairy products are the milk-based sources used (animal type, diet, age, lactation period, etc.) and food processing processes (temperature, storage conditions, etc.). In addition, starter cultures and/or probiotic types used in fermentation are a factor that directly affects the nutritional value of fermented milk products [24]. Probiotics are generally divided into three groups: LAB-based traditional probiotics, non-LAB probiotics, and next-generation probiotics. Although other microorganisms are used in fermented dairy products, LAB has a greater place. The main dairy products in which LAB are used fermented milk, yogurt, infant formula, cheese, butter and cream, and ice cream [25,26]. In general, dairy products are considered primary dietary sources for LABs as probiotics which can be found naturally or added afterwards [27]. Although there is no certain cell count level that can guarantee the health effects of the probiotic strain in a food product, at least 10^6^–10^8^ cfu/g is reported as a sufficient amount to benefit from the beneficial effects of probiotics [25]. This clearly shows that the presence of a culture that can show probiotic potentials does not guarantee that the product will be probiotic. Many factors affect the viability and stability of probiotics in dairy products. These are titratable acidity, pH, homogenisation, dissolved oxygen content, H_2_O_2_, storage temperature, type of probiotic, lactic acid concentration, and species and strains of the associative organism [28]. However, some methods have been developed to preserve the viability of probiotics. In this context, there are studies to increase probiotic viability with microencapsulation and prebiotic addition techniques [28,29].

**Figure 1:**
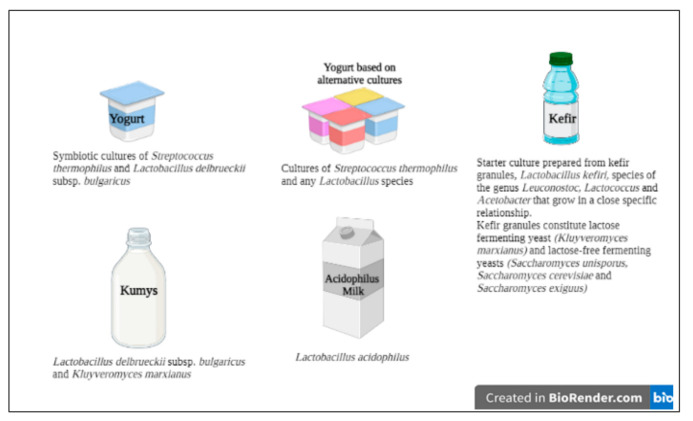
Specific starter culture(s) of some dairy products (adapted from Codex Alimentarius, 2011).

In fermented milk products, bacteria belonging to *Lactobacillus*, *Lactococcus*, *Leuconostoc*, *Pediococcus*, *Bacillus*, *Propionibacterium*, and *Bifidobacterium* genera are mostly prominent [30]. *Lactobacillus* genus is the largest genus with 314 species and has become the most associated keyword with probiotics in the last 20 years. However, there is no consensus among scientists about the acceptance of some LAB genus as probiotics [31]. Some of the most studied probiotic LAB in the literature are *Lactocaseibacillus rhamnosus* GG (ATCC53103), *Lactocaseibacillus rhamnosus* HN001, *Lacticaseibacillus casei* Shirota, *Lacticaseibacillus casei* Zhang, *Lactobacillus acidophilus* NCFM, *Lactobacillus acidophilus* LA-5, and *Lacticaseibacillus casei* DN-114 001. Recently, novel probiotic LAB such as *Limosilactobacillus reuteri* and *Lactobacillus johnsonii* are used in the development of functional dairy products [25,32]. According to the Codex Alimentarius definitions of milk and dairy products, the specific starter cultures of yogurt and other fermented milk products are given in Figure 1 [33]. Since the beneficial effects of probiotics are strain-specific, different strains of the same species can cause completely different effects on the host. Thus, it is stated that more studies are needed to understand the probiotic potential of new LAB strains and also well-known starter cultures of dairy products [34].

Some LAB strains isolated from dairy products, their potential bioactivities/probiotic potentials and stability issues are summarized in Table 2. LAB strains isolated from fermented dairy products are generally viable in low acidic conditions and various bile acid concentrations. However, some LAB have also been reported to show low stability under the same conditions [35]. In addition, many LAB have been reported to have the superb capability of adhesion to human intestinal cells and important auto-aggregation properties, as well as antimicrobial and immunomodulatory activities [36,37,38]. Available data support the high potential of LAB in novel functional food production. However, further studies are recommended to select the ideal combination and environment of probiotic strains as well as novel probiotic strains in the development of new probiotic products in the dairy industry.

As a result of significant research on human microbiota and probiotics in recent years, the gut has gained a new reputation as a “second brain” due to its microflora [46]. Undoubtedly, the discovery of the gut–brain axis underlies this. The gut–brain axis (HPA) is a bidirectional communication network that connects the enteric and central nervous systems. This network is not only anatomical but also includes endocrine, humoral/metabolic, and immune communication pathways [47,48]. This communication network includes the central nervous system (CNS), both the brain and spinal cord, the autonomic nervous system (ANS), the enteric nervous system (ENS), as well as the hypothalamic–pituitary–adrenal axis (HPA), thus connecting the gut and the brain to each other [11,47]. It is suggested that the communication network established between the gut and the brain in this way may be related to many important health parameters such as intestinal activities and gastrointestinal function, metabolic diseases, cognitive performance, and mental health [46,47].

The gut microbiota has the ability to modulate the ENS and CNS, with the ability to produce many neurotransmitters, as in the human brain [49]. Among the neurotransmitters produced locally by the gut microbiota, there are local neurotransmitters such as γ-aminobutyric acid (GABA), noradrenaline, dopamine, serotonin (5-hydroxytryptamine-5-HT), melatonin, histamine and acetylcholine, and a biologically active catecholamine form in the lumen, thereby can affect the nervous system activity [50,51,52]. This modulation is provided through many mechanisms including the vagus nerve and HPA and inflammatory cytokines as well as neurotransmitters [53]. In addition, the production of some molecules, such as nitric oxide, which interacts with the vanilloid receptor on capsaicin-sensitive nerve fibres by some specific microbes such as *Lactobacilli*, contributes to this modulation [54]. This modulation is influenced by genetics, lifestyle habits (nutrition, drug use, exercise, etc.), and many environmental factors (stress, fear, social interaction, etc.), and the composition of the intestinal microbiota plays a key role in this modulation [49]. In fact, as the evidence on the effectiveness of diet in the intestinal microbiota increases in recent years, the “gut–brain axis” network, known as bi-directional communication, will be started to be called three-directional as the “food–gut–brain axis” [48,55].

The limited studies on humans related to this axis are mostly conducted on LAB and are often associated with mental performance, psychological and related immunological parameters [47,56,57]. It was determined that a fermented milk product containing *Lactobacillus helveticus* IDCC3801 improved cognitive performance after 12 weeks in healthy older adults (60–75 years) [56]. In another study, the regular consumption of the milk product fermented with *Lacticaseibacillus casei* DN-114001 and yogurt culture for 6 weeks modulated the immune response (lymphocyte and CD56 cell count) of university students under academic stress was reported [57]. It is also found that the *Lactobacillus* increased after consumption of unpasteurized milk and products (without comparison with pasteurized milk as control), and microbiome profile and function and the level of faecal valerate, as determined by measuring estimated neuroactive potential using a gut–brain modulation approach are increased [12]. Thus, the results of all these researches showed that LAB originating from milk and dairy products play a key role in this axis and thus are related to health parameters. However, no correlation between LAB counts and mental and psychological measures was reported. Hence, it can be said that the evidence for a direct effect of dairy LAB on the gut–brain axis is still lacking 

## 4. Involvement of Dairy LABs in the Modulation of Non-Communicable Diseases (NCDs)

Milk and dairy products are consumed by more than 6 billion people worldwide, as they are a food group with a wide variety in terms of taste, texture, and nutritional value [58]. Although the number of studies supporting the beneficial effects of milk and its products on health, such as reducing body weight and waist circumference, decreasing the risk of Type 2 diabetes mellitus (T2DM), hypertension, and cardiovascular diseases (CVDs), has been increasing in recent years, there are studies in the literature that do not support these effects [59,60,61,62]. These inconsistent results are thought to be due to large differences in nutrient diversity among dairy products [58]. Many factors such as the fermentation of dairy products and the starter culture used in this fermentation process, the duration of fermentation, and the ambient temperature are responsible for this nutrient and flavour diversity [63].

**Figure 2:**
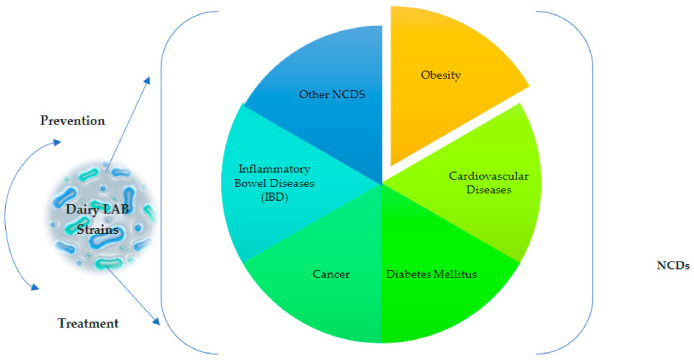
Non-communicable diseases (NCDs) reported to be possibly modulated by some dairy LAB strains.

Fermented dairy products may show their health-promoting effects due to the influences of microbial metabolites (biogenic or bioactive effect) formed during the fermentation process, as well as sometimes the probiotic effects of the certain LAB strains isolated from their composition [64,65]. It is stated that the most important bioactive compounds formed by LAB activity during the fermentation process are peptides, exopolysaccharides (EPS), bacteriocins, some amylase, protease, lipase enzymes, and lactic acid [66]. However, not every LAB strain can produce all of these compounds. While some LAB strains show antihypertensive effects by producing ACE inhibitory peptides, some strains show anti-diabetic, cholesterol-lowering, anti-tumoral, and immunomodulatory effects by producing EPS [66,67,68]. For this reason, it is of great importance to examine dairy LAB strains, which are effective in preventing NCDs and supporting their treatment. In this regard, NCDs are associated with dairy LAB strains and are thought to be modulated by these bacteria (Figure 2).

### 4.1. Obesity

Obesity is defined as abnormal or excessive fat accumulation that can impair health and results from a number of factors, such as decreased energy expenditure and increased energy intake [69,70]. It is a serious public health problem in developed and developing countries around the world, and its prevalence is increasing day by day [71]. The World Health Organization (WHO) states that the prevalence of obesity has nearly tripled since 1975 [70]. Common obesity-related comorbidities such as T2DM, hypertension, and heart disease have an impact on the social and financial system [71,72]. For this reason, the treatment and prevention of obesity by applying effective treatment methods is very important for both the health and quality of life of individuals and the economy of countries. Similarly, research on foods and nutrients that can protect against obesity and support its treatment has increased in recent years. It is stated that fermented milk products, which are among these foods, show these effects by the probiotic potential of the certain starter LAB strains used in fermentation and the formation of various biogenic metabolites (EPS, a bioactive peptide, etc.) [64].

Protein hydrolysates formed as a result of LAB activity can demonstrate a stimulating effect on the intestinal hormones such as glucagon-like peptide 1 (GLP-1), glucose-dependent insulinotropic polypeptide or gastric inhibitory peptide (GIP), and cholecystokinin (CCK), which are secreted in the intestine in response to food intake and also act as satiety signals [73]. In a study comparing mice on a high-fat diet (HFD) and consuming fermented whey beverage (*Lactiplantibacillus plantarum* DK211 and *Lactococcus lactis* cultured) in addition to HFD, food intake, blood glucose, serum insulin, leptin, and ghrelin levels of those who consumed whey beverage were found to be significantly lower compared to those who only consumed HFD [72].

Obesity and dietary high fat consumption cause an increase in the firmicutes/bacteroidetes ratio and the development of dysbiosis, leading to increased intestinal permeability, metabolic endotoxemia, adipose tissue inflammation, and metabolic diseases [74,75,76]. In addition, the increase of pathogenic bacteria in the intestinal microflora stimulates the production and the secretion of lipopolysaccharide (LPS) from epithelial cells [75]. LPS can lead metabolism to inflammatory processes by binding to cytokine receptors that trigger the release of pro-inflammatory cytokines on adipocytes. Dairy LAB strains with probiotic properties can compete with pathogenic bacteria and reduce these effects of dysbiosis and inflammation-related gene expression [75]. Kadooka et al. showed that consumption of fermented milk containing LG2055 at doses of 10^6^ and 10^7^ cfu/g/day in individuals with visceral obesity resulted in significant reductions in visceral adiposity, body mass index (BMI), body fat mass, waist, and hip circumference [77]. This effect of LG2055 is explained by its recognition by intestinal epithelial cells (IEC) and supporting anti-inflammatory and epithelial integrity protective mechanisms of IEC [78]. Bacteriocins produced by LAB have potential anti-obesity effects by showing antimicrobial effects on bacteria in the intestinal microbiota that cause dysbiosis [79].

LAB strains isolated from kefir culture, especially the *Lactobacillus kefiri* DH5 strain, reduced body weight and adiposity in mice fed with HFD and maintained their viability throughout the gastrointestinal system (GIS)[80]. It has been determined that *Lactobacillus kefiri* DH5 exerts these anti-obesity effects by reducing cholesterol absorption in the intestinal lumen and increasing the expression of PPARα, carnitine palmitoyl transferase-I (CPT1), and fatty acid-binding protein 4 (FABP4) in adipose tissue. To further elucidate this mechanism, as the CPT1 and FABP4 genes are mediated by PPARα, increased PPARα activity induces upregulation of these two target genes [80]. CPT1 is an enzyme located in the mitochondrial outer membrane that catalyses the transport of acyl groups for β-oxidation of fatty acids [81]. FABP4 is an intracellular protein that suppresses lipogenesis and promotes lipolysis in adipose tissue. As a result, consumption of kefir containing *Lactobacillus kefiri* DH5 upregulates the expression of CPT1 and FABP4 genes, thereby can increase β-oxidation of fatty acids and lipolysis and can decrease lipogenesis in metabolism and may have an anti-obesity effect [80].

Dairy LAB suppresses the pancreatic lipase enzyme, thus preventing the absorption of fats for use as an energy source and increasing their faecal excretion. It has been stated that products containing bacteria with this inhibitory feature can be used for the treatment of obesity [82]. It was determined that various strains of *Lacticaseibacillus* *casei*, *Limosilactobacillus fermentum*, and *Lactobacillus helveticus* were able to inhibit pancreatic lipase by 2–25%. *Lacticaseibacillus* *casei* (NK9) strain isolated from Dahi, a fermented milk product, showed the highest inhibition [82]. However, quite different results were obtained even among different strains of the same bacterial species.

Anti-obesity effects may also occur as a result of the biological effects of EPS produced by some dairy LAB strains during the fermentation process. Kefiran, a water-soluble, heteropolysaccharide EPS produced by *Lactobacillus kefiranofaciens* found in kefir grains, is one of the dairy EPS whose biological effects have been most studied. Mice that consumed kefir-isolated EPS in addition to a high-fat diet had significantly lower body weight and fat gains than those who received a high-fat diet alone [83]. The anti-obesity effect of EPS is generally associated with its viscous and water-soluble structure. However, the anti-obesity effects of EPS were found to be significantly better when the weight and fat loss of the EPS group and those given beta-glucan with higher viscosity were compared [83]. In addition, it was determined that the *Akkermansia* genus increased in faeces in the group receiving EPS supplementation [83]. It is stated that *Akkermansia* species have probiotic effects that protect intestinal integrity and prevent obesity by increasing the mucin layer thickness and goblet cell thickness and decreasing metabolic endotoxemia [84]. In other words, in addition to its viscosity and volume-increasing effect, its potential prebiotic properties also have an important place in the anti-obesity mechanism of EPS [83]. EPS produced by dairy LAB strains can show these prebiotic effects by passing through the colon without being hydrolysed by human digestive enzymes, making direct contact with the intestinal epithelium, and producing metabolites with biological activity [85].

It is also indicated that dairy LAB strains support the anti-obesity effect by increasing the amount of conjugated linoleic acid (CLA) in the composition of the milk during the fermentation process [86]. CLA has a weight-reducing effect by decreasing food intake, proliferation, adipocyte differentiation, and lipogenesis, and by increasing lipolysis, β-oxidation of fatty acids, and energy expenditure [86].

### 4.2. Cardiovascular Diseases

Cardiovascular diseases (CVD) are one of the leading causes of death in the world, and their prevalence is increasing day by day. They are also the most common cause of death, accounting for an estimated 17.9 million deaths each year (31% of all deaths) [87]. American Society for Preventive Cardiology (ASPC) indicated that the most important risk factors of CVD are unhealthy diet, physical inactivity, dyslipidaemia, hyperglycaemia, hypertension, obesity, individual differences between patients (age, race/ethnicity, and gender), thrombosis/smoking, renal dysfunction, and genetic/familial hypercholesterolemia [88]. Studies conducted in recent years stated that there is a negative relationship between the consumption of fermented milk products and the risk of CVD [59,62]. In post-MI patients, each 25 g/d increase in yogurt consumption, regardless of fat content, was found to reduce the CVD-related mortality risk by 4% and the overall mortality risk by 2%, while no such association was observed in other dairy products such as milk and cheese [89].

One of the most important risk factors playing a role in the pathophysiology of CVD is hypercholesterolemia. Clinical studies have shown that regular administration of probiotic *Lactobacillus* strains can have hypocholesterolaemic effects [90]. LAB and bifidobacteria isolated from human intestines can show a cholesterol assimilation effect, and that reduction of cholesterol content in the body by 1% by LAB, accounts for reduced incidence of CVD by 2% to 3% [91]. In addition, it has been reported that regular consumption of a fermented milk product containing *Lactobacillus acidophilus* L1 strain provides a 2.9% reduction in blood cholesterol; hence, it has the potential to reduce the risk of coronary heart disease by 6 to 10% [92]. Moreover, following the consumption of milk fermented with *Lacticaseibacillus paracasei* subsp. *paracasei* NTU 101, *Lactiplantibacillus plantarum* NTU 102, and *Lactobacillus acidophilus* BCRC 17,010 strains were found to be effective in reducing total cholesterol, low-density lipoprotein cholesterol (LDL-C), high-density lipoprotein cholesterol (HDL-C), and triglyceride levels of hyperlipidaemic mice fed a high-cholesterol diet [93].

LAB showing probiotic properties basically have cholesterol-lowering effects through mechanisms including cholesterol assimilation, binding/addition of cholesterol to cellular components such as cell surface or membrane, enzymatic deconjugation of bile acids with bile salt hydrolase (BSH), suppression of de novo synthesis of cholesterol by short-chain fatty acids (SCFA) produced by probiotics [94,95]. However, not all LAB strains in fermented milk products show a hypocholesterolaemic effect. Yogurt fermented with the use of *Streptococcus thermophilus* and *Enterococcus faecium* strains together provided a significant decrease in serum cholesterol levels (8.4%), while yogurts produced with the use of *Streptococcus thermophilus* and *Lactobacillus acidophilus* or *Lactocaseibacillus rhamnosus* strains together or alone did not show a hypocholesterolaemic effect [96]. As a result of the examination of 58 potential probiotic LAB strains for their in vitro survival in digestion and cholesterol-lowering abilities, the best-performing strains were *Lacticaseibacillus casei*, *Lacticaseibacillus paracasei*, *Lactiplantibacillus plantarum*, *Enterococcus faecium*, and *Enterococcus lactis* with some strains providing reduction of cholesterol levels between 42 and 55% in the broth [97]. In another study, after the comparison of the change in lipid profiles of individuals consuming probiotic yogurt (*Streptococcus thermophilus*, *Lactobacillus bulgaricus*, *Lacticaseibacillus casei* subsp. *casei*; 10^8^ cfu/g) and normocholesterolemic women consuming traditional yogurt (*Streptococcus thermophilus*, *Lactobacillus bulgaricus*; 10^8^ cfu/g), no significant difference was observed between the groups. Therefore, regular consumption of both probiotic and traditional yogurt for 4 weeks has been shown to have a positive effect on the lipid profile (total/HDL and LDL/HDL cholesterol ratio) in the plasma of healthy women [98].

Oral administration of *Lacticaseibacillus casei* Shirota (LcS), a probiotic dairy LAB strain, has been shown to ameliorate obesity-associated metabolic abnormalities such as insulin resistance, impaired glucose tolerance, type 2 DM, hepatic steatosis, and hyperlipidaemia in animal models [99,100,101]. In obese pre-diabetic individuals consuming milk fermented with LcS YIT 9029, this dairy LAB strain reduces dietary cholesterol absorption by binding and/or assimilating sterols and has the potential to prevent hypercholesterolemia individuals who consumed 100 mL/day of fermented milk for 8 weeks had significantly lower total LDL, and non-LDL cholesterol levels compared to the placebo group [102].

Some LAB strains have bile salt hydrolase (BSH) enzyme, and this enzyme catalyses the hydrolysis of conjugated bile salts to free bile salts and amino acid residues [103,104]. Expression of BSH in the intestinal lumen converts conjugated bile salts, which have high solubility and absorption, into free bile salts with low solubility. Therefore, the reabsorption and recycling of bile salts decrease, and the de novo synthesis of bile salts from cholesterol in the liver increases. This synthesis requires cholesterol transport from the blood to the liver, thereby lowering total serum cholesterol levels [103]. For example, dairy products fermented with *Lactiplantibacillus* *plantarum* SC70 and SC80 strains can reduce cholesterol levels by showing high lipase inhibition (>35%) and BSH activity [103]. Moreover, as a result of adding *Lactiplantibacillus* *plantarum* GKM3 on a high-fat diet of mice for 6 weeks, total lipid, cholesterol, and triglyceride levels in the liver decreased, and faecal lipid, cholesterol, and triglyceride excretion increased significantly [105]. The increase in faecal cholesterol and lipid excretion suggests the effect of lipase inhibition and BSH enzyme activity.

Another mechanism by which dairy LAB is effective in regulating blood cholesterol levels is suppressing cholesterol synthesis by inhibiting the 3-hydroxy 3-methyl glutamyl CoA (HMG-CoA) reductase enzyme. *Lactobacillus acidophilus*, one of the dairy LAB species, inhibits HMG-CoA reductase, a rate-limiting enzyme responsible for endogenous cholesterol biosynthesis in the body, and this enzyme may cause a decrease in cholesterol concentration by deconjugating bile acids in the intestine [106].

However, some bacteria attach to the cholesterol surface, causing the cholesterol to become less available for absorption. In a study, when the cholesterol-binding properties of the active and dead cells of *Lactobacillus kefiri* JK17 isolated from kefir were compared, it was observed that no cholesterol was bound to the dead cell membrane, while a small amount of cholesterol was bound in the active cells [107]. Although there are not many studies related to this mechanism, the results of the current study suggested that dairy LAB should maintain their viability in the intestine to show cholesterol-binding properties.

Oxidative modification of LDL plays a crucial role in the initiation and progression of atherosclerosis. LDL has cytotoxic potential, which is responsible for endothelial cell damage and macrophage degeneration in atherosclerotic plaques [108]. For this reason, malondialdehyde (MDA)-LDL and/or MDA-LDL/LDL-cholesterol levels, which are the indicators of LDL oxidation, may be a good risk indicator for CVDs [109,110]. A double-blind, placebo-controlled study examined the effects of consumption of fermented milk containing *Streptococcus thermophilus* YIT 2001, which has high anti-oxidative activity, on LDL oxidation and blood pressure. It was observed that the decrease in serum MDA-LDL, MDA-LDL/LDL-cholesterol, systolic blood pressure (SBP), and diastolic blood pressure (DBP) values of the fermented milk group were found to be significantly higher than the control group (skimmed milk) [108]. In a different study, oxidative stress and MDA levels decreased, while serum GSH-Px, SOD, and CAT levels increased as a result of exposure to high doses (50 mg/kg per day) of EPS formed by *Lactiplantibacillus* *plantarum* YW11 in aged mice [90]. EPS production may reduce hyperlipidaemia by showing a cholesterol-binding effect as well as supporting the antioxidant defence. A study comparing the cholesterol-binding abilities of EPS-producing and non-EPS producing *Lactococcus* *lactis* subsp. *cremoris* showed that the EPS-producing strain could bind more cholesterol than the non-EPS producing strain. It has been stated that these polysaccharides can reduce blood cholesterol levels by binding cholesterol, reducing its absorption, and increasing faecal excretion, similar to soluble dietary fibre [111].

Hypertension is a chronic degenerative disease characterized by blood pressure values exceeding normal limits [112]. In addition, it is an important risk factor for the development of other CVD, strokes, renal failure, cerebrovascular accidents, and many other complications [113]. The Framingham heart study reported that a 2 mmHg reduction in DBP was associated with a 17% reduction in the prevalence of hypertension, a 15% reduced risk of stroke, and a 6% reduced risk of coronary heart disease [114]. The ACE has a key role in the control of blood pressure and converts Angiotensin I to angiotensin II, which has vasoconstrictor properties and hydrolyses vasodilator peptides such as bradykinin and kallidin, causing an increase in blood pressure [113]. Inhibition of this enzyme increases the vasodilator response and shows a blood pressure-reducing effect. It is stated that the bioactive peptides formed by the LAB in the fermentation process in fermented milk products have a blood pressure regulatory effect by inhibiting the ACE enzyme. These bacteria hydrolyse the proteins in milk composition by showing a proteolytic effect and form ACE inhibitor oligopeptides such as Val-Pro-Pro (VPP) and Ile-Pro-Pro (IPP). At the same time, it can produce hypotensive peptides such as casokinins and lactokinins due to its proteolytic activity [1,64]. It was determined that consuming milk fermented with *Lactococcus* *lactis* NRRL B-50571 and consuming captopril (an ACE inhibitor) for 6 weeks had similar effects on SBP and DBP, and these values were significantly different from the control group. In addition, at the end of 6 weeks, the SBP value of the group consuming fermented milk products decreased by 49.9 ± 14.2 mmHg, while those consuming captopril decreased by 45.2 ± 23.6 mmHg. The plasma ACE level was 5.5 times lower, and the plasma nitric oxide (NO) level was 1.6 times higher in those who received fermented milk and captopril compared to the control group [115]. NO is an important bio-regulatory molecule that improves endothelial function and vasodilation and controls blood pressure. Increased inflammatory processes and oxidative stress in the body decrease endothelial NO synthase (eNOS) enzyme activity, leading to endothelial dysfunction, decreased NO bioavailability, and consequently may hypertension [116]. In a study, as a result of the application of VPP and IPP for 5 days, it was determined that the significant induction of the eNOS gene (1.89-fold) increased the NO levels, caused a slight increase in the expression of the cyclooxygenase (COX-1) gene, and caused a decrease in the expression of the NF-kB and PPARγ genes [117]. A double-blind, randomized controlled trial evaluating the blood pressure lowering effects of *Lactococcus lactis* NRRL B-50571 in prehypertensive patients found after 5 weeks, systolic (116.55 ± 12.26 mmHg vs. 124.77 ± 11.04 mmHg) and diastolic blood pressures (80.7 ± 9 vs. 84.5 ± 8.5 mmHg) were significantly lower in those who consume fermented milk compared to control group [118]. Even if SBP and DBP values at the end of the intervention were significantly decreased compared to baseline in those consuming fermented milk, this effect disappeared 1 week after the intervention was discontinued [118]. In another study, hypertensive rats consumed milk fermented with *Lactococcus lactis* NRRL B-50572 and *Lactococcus lactis* NRRL B-50571 strains, and capoptil, and water for 4 weeks. As a result, SBP and DBP values of the group consuming *Lactococcus lactis* NRRL B-50571 FM were significantly lower compared to groups consuming *Lactococcus lactis* NRRL B-50572 and water, while SBP values of a group consuming *Lactococcus lactis* NRRL B-50572 and a group consuming water were found to be similar. However, at the end of the intervention, it was seen that the lowest LDL-C and TG values were in the group consuming *Lactococcus lactis* NRRL B-50572 FM [119]. Dyslipidaemia may also play a role in the development of hypertension by causing endothelial damage [115]. Since it can be concluded that by different mechanisms, both *Lactococcus lactis* strains have antihypertensive properties and exert protective effects against CVD. However, it is not very accurate to generalize this result for all *Lactococcus lactis* strains. In a study examining the proteolytic, antioxidant, and ACE inhibitor activities of milk fermented with *Lactobacillus helveticus* NK1, *Lactocaseibacillus rhamnosus* F, and *Limosilactobacillus* *reuteri* LR1 strains, the antioxidant activity increased depending on the proteolytic activity of the bacterial strain whereas there was no such association between the ACE inhibitor and proteolytic activities. In addition, although the proteolytic activity of *Lactobacillus helveticus* NK was 2 times higher than that of *Lactocaseibacillus rhamnosus* F, ACE inhibitions of these strains were found to be similar. Moreover, when the peptide profiles of fermented milk samples were examined, the ACE inhibitory property was associated with peptides consisting of the C-terminus of αS2-casein [48]. The strong antihypertensive and ACE inhibitory properties of peptides produced from caseinates and casein fractions have been associated with ionic calcium (Ca^2+^) in their structure [120].

However, in some cases, the consumption of ACE inhibitory peptides or fermented products containing the bacteria that compose them does not show ACE inhibitory effects. This was explained by the fact that ACE inhibitor peptides are not able to act in target organs due to their further degradation during gastrointestinal digestion [115]. In addition, processes to be applied to the milk to be fermented may cause a similar effect by decreasing serum albumin, β-lactoglobulin, and α-lactalbumin concentrations and leading to a change in the structure of proteins. Excessive thermal and alkaline processing of milk can prevent the formation of bioactive peptides, which are ACE inhibitors and have antioxidant properties, by forming intermolecular covalent bonds that are resistant to the action of hydrolytic enzymes leading to indigestible peptide bonds [64]. The use of sterile milk and milk heated up to 90 °C had a protective effect on health (Table 3).

### 4.3. Diabetes Mellitus

Diabetes is defined as a global epidemic with serious health, social, and economic impacts. In the past three decades, the prevalence of T2DM has more than doubled [133]. The global T2DM prevalence was 9.3% (463 million people) in 2019, and it is estimated that this rate will increase to 10.2% (578 million people) by 2030 and to 10.9% (700 million people) by 2045 [134]. T2DM is more common in adults and accounts for about 90% of all diabetes cases. Diabetes mellitus is a chronic disease characterized by hyperglycaemia caused by resistance to insulin or insufficient secretion of this hormone by pancreatic β cells [135]. Experiments from different parts of the world have revealed that lifestyle modification with physical activity and/or healthy nutrition can delay or prevent the development of T2DM [136]. Studies examining the association between the consumption of dairy products and the incidence of diabetes indicate an inverse association, although there are no consistent results [137,138,139]. The inconsistency of the results is explained by the fact that dairy products are not analysed according to their subgroups (fatty, fat-free, fermented, etc.). Among dairy products, especially low-fat and fermented milk products have been shown to reduce the incidence of diabetes in various studies [139,140]. These effects are achieved as a result of dairy LAB strains showing probiotic properties and forming bioactive peptides, thereby reducing oxidative stress, regulating intestinal microflora, showing immune-modulatory and anti-inflammatory effects [141].

FFAs, adipokines, and various pro-inflammatory cytokines released from adipose tissue have an important role in the pathophysiology of T2DM. Increased circulating free fatty acids and their oxidation products may activate the serine/threonine kinase cascade, resulting in impaired insulin signalling [142]. To compensate for this defect in insulin signalling, more insulin secretion from pancreatic β cells results in endoplasmic reticulum stress, protein misfolding, and ultimately apoptosis in β cells [142]. *Lactobacillus acidophilus*, one of the dairy LAB strains, inhibits the production of reactive oxygen metabolites and cytokines responsible for damage to pancreatic cells and has a regulatory effect on serum insulin levels [106]. Fasting blood glucose, insulin, glycosylated haemoglobin (HbA1c), and circulating free fatty acid levels of rats in those consuming fermented milk product (Dahi) containing probiotic bacteria (*Lactobacillus acidophilus*, *Lacticaseibacillus casei*, and *Lactococcus* *lactis biovar diacetylactis*) with a high fructose diet for 8 weeks were significantly lower compared to those only fed a high fructose diet, and it was similar to those fed a normal diet [143].

As a result of the increase in Gram-negative bacteria causing dysbiosis in the intestine and the deterioration of intestinal barrier function, the circulating level of LPS associated with these bacteria increases. An increase in LPS level, however, activates the NF-kB pathway, increasing the circulating level of pro-inflammatory biomarkers (CRP, TNF-α, IL-1β, and IL-6) and plays a role in the pathogenesis of insulin resistance in patients with Type 2 DM [106,144,145]. It was reported that when elderly individuals with small intestinal bacterial overgrowth (SIBO) consume 300 g of 10^9^ CFU of *Lactobacillus johnsonii* La1-containing yogurt daily for 4 weeks, a decrease in plasma LPS pattern recognition receptors and endotoxin levels has been detected [145]. Dairy LAB strains can exert a protective effect on pancreatic cells by inhibiting the production of these inflammatory cytokines [146]. A study determined that fasting blood glucose and serum IL-6 levels were significantly reduced in slightly obese individuals consuming yogurt containing *Lactiplantibacillus* *plantarum* OLL2712 (>5 × 10^9^ cells/112 g of yogurt) [147]. It was shown in individuals with type 2 DM that consumption of 300 g of probiotic yogurt (*Lactobacillus delbrueckii* subsp. *bulgaricus* and *Streptococcus thermophilus*, *Bifidobacterium animalis* subsp. *lactis* Bb12 (DSM 10140) and *Lactobacillus acidophilus strain* La5; 3.7 × 10^6^ cfu/g) provides a decrease in HbA1c and TNF-α levels while it does not cause a significant change in fasting blood glucose, IL-6, hs-CRP values [146]. These different results between studies may be due to differences among LABs strains added to yogurt. However, it can still be concluded that probiotic yogurts containing LAB strains have anti-inflammatory and antidiabetic effects, but on different pro-inflammatory markers.

In the case of T2DM or insulin resistance, the failure of insulin-dependent glucose uptake into fat and muscle tissues results in increased blood glucose concentrations and increased glucose uptake into insulin-independent tissues. This leads to an increase in oxidant products such as advanced glycation end products (AGEs), and with the activation of macrophages, increasing inflammatory cytokines and oxidative stress, and causing β-cell damage [106,146]. Fermented kinds of milk are among the natural antioxidant sources of the diet owing to the antioxidant peptides in their composition. It has been stated that αs-casein-derived bioactive peptides, one of the most important bioactive peptides, have free radical scavenging, enzymatic and non-enzymatic lipid peroxidation inhibitory effects. Moreover, it has been suggested that antioxidant peptides derived from whey protein have intracellular antioxidant properties since cysteine-rich amino acids in their structures help glutathione synthesis [141]. In a study, the consumption of *Lactobacillus mali* APS1 strain (5 × 10^8^–10^9^) isolated from sugary kefir in rats fed a high-fat diet for 12 weeks increased the number of butyrate-producing bacteria in the intestinal microbiota and GLP-1 activation, thereby resulting in upregulated SIRT-1 and PGC-1α and downregulated SREBP-1. As a result of these, it was observed that blood glucose was regulated, HOMA-IR values were decreased, and lipid metabolism was regulated. Moreover, SIRT-1 upregulation also suppressed hepatic oxidative stress by increasing Nrf2 activation and antioxidant enzyme production in the liver [148]. Another study also observed that kefir intervention activates antioxidant enzymes through the Nrf2 pathway in diabetic individuals [149]. In another study related to the change in antioxidant activities of 25 LAB strains during milk fermentation, *Leuconostoc mesenteroides* subsp. *cremoris*, *Lactococcus* *lactis*, *Lactobacillus acidophilus*, *Lactobacillus jensenii,* and *Lactobacillus helveticus* strains inhibit radical scavenging activity and lipid peroxidation, and this activity increases during the fermentation process. It was observed that the bioactive peptides that emerged during the fermentation process were effective in the antioxidant activity of *Leuconostoc mesenteroides* subsp. *cremoris Lactobacillus jensenii*, and *Lactobacillus acidophilus* strains. However, it has been determined that not every bioactive peptide has this effect. For example, *Lactobacillus helveticus*, which has high proteolytic activity, exerts a moderate level of antioxidant properties. In addition, fermented milk products using mixed LAB culture showed higher radical scavenging activity than those using a single bacterial strain [150].

Dietary carbohydrates are hydrolysed from the small intestine by α-amylase, pancreatic amylase, and α-glucosidase enzymes, thus regulating blood glucose. It has been stated that LAB colonizing the intestinal epithelium can reduce glucose absorption and help regulate blood glucose by using the glucose formed due to hydrolysis as an energy source [146]. Moreover, inhibition of pancreatic amylase and α-glucosidases may be an effective treatment method in T2DM [151]. When assessing the α-amylase suppressive effects of plain and fruity yogurts of various brands, it was determined that the α-amylase suppressive effect of plain yogurts could be up to 68% depending on the brands [151]. Furthermore, it has been stated that milk fermented with *Limosilactobacillus fermentum* M2 and M7 starter cultures showed a suppressive effect on α-amylase (65.3% and 63.5%, respectively) and α-glucosidase (11.3% and 13.7%, respectively) enzymes. The main reason for this effect was peptides consisting of milk proteins such as whey and casein in the fermentation process [82]. The bioactive peptides formed by the proteolytic activity of LAB strains have been shown to have anti-DM effects with different mechanisms. For example, as a result of the proteolytic activity of *Lactococcus lactis*, it was stated that bioactive oligopeptides consisting of α- and β-caseins stimulate the release of insulin and somatostatin and delay the gastrointestinal absorption of nutrients [1]. In another study, it was observed that HOMA-IR, blood glucose, and fasting insulin levels of mice treated with kefir peptide were significantly reduced compared to the control group [152]. Therefore, it has been suggested that kefir peptides treatment can improve glucose tolerance and reduce insulin resistance [152].

Adiponectin is an adipocyte-derived serum protein that plays an important role in increasing insulin sensitivity [153]. Adiponectin regulates many metabolic processes including the maintenance of energy balance, glucose homeostasis, and lipid metabolism, and adiponectin levels are generally lower in obese individuals [127]. It was found that obese rats fed yogurt fermented with *Limosilactobacillus fermentum* TSI with the high-fat diet had significantly higher adiponectin levels than those fed the high-fat diet alone [127].

The connection between dairy consumption and DM in the literature mostly compared traditional yogurts with probiotic yogurts containing strains such as *Lactobacillus acidophilus* La5 and *Bifidobacterium lactis* Bb12 [106,141,146]. As a result of these studies, it was observed that probiotic yogurts were significantly more effective on diabetes-related parameters such as blood glucose, HbA1c, TNF-α, antioxidant capacity [141,146]. However, traditional yogurts containing only LAB strains (*Lactobacillus bulgaricus* and *Streptococcus thermophilus*) did not show any improvement in diabetes-related parameters [106,141,146].

### 4.4. Cancer

Cancer is defined as the proliferation of malignant cells resulting from genetic and epigenetic mutations. According to WHO data, cancer is the second leading cause of death globally [154]. Most types of cancer are caused by genetic changes or damage that builds up in cells over time. This damage can be caused by individual characteristics such as inherited mutations, hormonal characteristics, immune status, and environmental factors such as smoking, infectious organisms, chemicals, and dietary components [155]. Observational studies have indicated that approximately 30–40% of cancer cases can be prevented by regulating nutrition-related factors [156]. Although a consistent association cannot be obtained between the consumption of dairy products and the risk of cancer owing to the wide variety of foods in dairy products, it is suggested that the LAB in fermented milk products and their bioactive compounds and calcium in the composition of milk are effective in reducing the risk of cancer [157,158]. For example, probiotics such as *Lactobacilli* and *Bifidobacteria* found in fermented milk products reduce the risk of colon cancer by changing the activity of faecal enzymes such as glucuronidase, β-glucuronidase, azortortase, nitroreductase, and increasing immune cell activity [159]. β-glucuronidase is an enzyme responsible for the hydrolysis of glucuronides in the intestinal lumen. This reaction produces toxic and carcinogenic substances that are detoxified by the formation of glucuronides in the liver and then enter the intestine via bile [159]. Toxic compounds in the aglycone structure can be produced in the intestine by bacterial β-glucuronidases. In humans, faecal β-glucuronidase activity is higher in colorectal cancer patients than in healthy controls, suggesting that this enzyme plays a role in carcinogenesis [159]. Another enzyme important in the prevention of colon cancer is nitroreductase. This enzyme is responsible for the reduction of nitro compounds to aromatic amines. Highly reactive intermediates and end products obtained from these reactions, such as reactive nitroso, N-hydroxyintermediates, and aromatic amines show mutagenic and carcinogenic effects. The reduction of aromatic nitro and azo compounds can be achieved by the activity of intestinal flora [159].

Probiotic LAB found in dairy products can prevent intestinal carcinogenesis by causing some changes in the intestinal microbiota. Probiotic LAB show these effects by inhibiting the growth of bacteria that convert pro-carcinogens to carcinogens in the intestinal microbiota and by reducing the number of carcinogens in the intestine [160]. The effects of *Lactobacillus kefiri* were investigated in some types of cancer (gastric cancer cells, breast cancer cells, and human peripheral blood mononuclear cells). It has been shown that this LAB strain induces apoptosis in gastric cancer cells, and apoptosis is associated with decreased polarization of mitochondrial membrane potential and decreased Bcl2 expression. However, in other cancer cells, *Lactobacillus kefiri* has not induced apoptosis. In this context, it was emphasized that this strain might be a potential agent in the treatment of gastric cancers [161].

Studies are showing that dairy LAB also have beneficial effects on breast cancer as well as digestive system cancers [128]. A recent study found that administration of milk fermented with a strain of *Lacticaseibacillus* *casei* modulated the immune response to breast cancer tumour in a mouse model, where tumour growth was significantly slowed or blocked in the fermented milk group compared to the control group (unfermented milk) [131]. It was reported that milk fermented with *Lactobacillus helveticus* R389 slowed the growth of breast tumour. This fermented milk has been found to increase interleukin (IL)-10 and IL-4 while decreasing IL-6 in serum, mammary glands, and immune cells [160]. High IL-6 systemic levels are correlated with poor prognosis in breast cancer patients and may be a biomarker for tumour burden [130]. In a study about the effects of fermented milk containing *Lacticaseibacillus* *casei* CRL431 strain on breast cancer in BALB/c mice, fermented milk delays tumour development, reduces pro-inflammatory markers, improves immune response, and provides some positive changes at the cellular level (Table 3) [128,129].

Cancer patients often receive treatments such as chemotherapy and radiotherapy that can reduce their quality of life and compromise their immune system. Although studies with dairy LABs seem promising, the efficacy and safety of probiotics used in these products should be evaluated under specific conditions [129]. However, there are not enough human studies yet, in which the application of probiotics as a bio-therapeutic against cancer is tested [131].

### 4.5. Inflammatory Bowel Diseases (IBD)

IBD is a non-infectious chronic inflammatory disease of the gastrointestinal tract. According to their clinical features, it includes two chronic idiopathic diseases, ulcerative colitis (UC) and Crohn’s disease (CD) [162]. Although IBD is a global disease and its prevalence is increasing worldwide, its pathology is not yet understood. Recent data have suggested that genetic factors, gut microbiota, and environmental factors play a role in the pathogenesis of this disease. Europe and North America are reported as the regions with the highest prevalence of IBD [163]. In recent years, the incidence of IBD has been increasing in countries that have just started to industrialize [164]. Although clinical studies are limited, it is reported that fermented foods may have positive effects on IBD symptoms. It is thought that especially fermented foods containing LAB can positively affect IBD by positively changing intestinal microbiota [165,166]. In this context, it was reported by Saez-Lara et al. (2014) that LAB and bifidobacteria found in dairy products and expressed as probiotics may have beneficial effects on the prevention and treatment of IBD by changing the intestinal microbiota [166].

Inflammatory processes, particularly Th1 and Th17 cells, are known to play a role in the pathophysiology of IBD. In a study, the effect of fermented milk on the Th1/Th17 response was evaluated in a murine model with mild IBD. Milk fermented with *Limosilactobacillus* *fermentum* has been shown to reduce the inflammatory response at week 6 through the metabolites or cell components it contains (especially LAB and cell components) [167]. Probiotic LAB may exert anti-inflammatory effects through some possible pathways at the cellular level by down-regulating pro-inflammatory cytokines such as IL-1β, IL-6, IL-17, TNF-α, and IFN-γ [168,169]. In addition, bioactive peptides can provide anti-inflammatory activity by down-regulating LPS-induced cytokine production in monocyte cells via the NF-kB pathway [170]. To observe anti-inflammatory effects of probiotic yogurt in IBD patients, participants were given yogurt containing *Lactocaseibacillus* *rhamnosus* GR-1 and *Limosilactobacillus reuteri* RC-14 for 30 days. Probiotic yogurt intake has been associated with significant anti-inflammatory outcomes in IBD patients and similar the expansion of the peripheral pool of putative regulatory T (T_reg)_ cells [165].

It has been shown that kefir and kefir fractions have anti-inflammatory and antioxidant activities, and kefir has a healing effect on dextran sulphate sodium (DSS)-induced colitis in rats[171]. Sevencan et al. (2019) reported that 10 mL of kefir daily relieves clinical signs and colonic macroscopic damage in rats with colitis caused by trinitrobenzene sulfonic acid. Still, a daily dose of 30 mL kefir has been shown to exacerbate colitis in the same study. Therefore, the effect of kefir (*Lactococcus* *lactis* subsp., *Leuconostoc* subsp., *Streptococcus thermophilus*, *Lactobacillus* subsp., and kefir yeast) to ameliorate colitis-induced symptoms is dose-dependent [172]. To investigate the immunomodulatory effects of *Lactobacillus kefiri* CIDCA 8348 strain isolated from kefir on intestinal T cells from patients with active IBD, the presence of *Lactobacillus kefiri*, the proliferation rate of lamina propria T cells was low, and the secretion of TNF-α, IL-6, IFN-γ, and IL-13 decreased. In addition, *Lactobacillus kefiri* induced an increased frequency of CD4+ FOXP3+ Lamina Propria T Cells (LPTC) with high IL-10 levels. These findings suggest that *Lactobacillus kefiri* strain CIDCA 8348 may have immunomodulatory effects on intestinal cells of IBD patients [173].

## 5. Conclusions

Dairy products especially those fermented ones are the valuable source of dairy LAB. These microorganisms are responsible for beneficial responses on the host by acting at different levels that include the production of bioactive compounds into the food matrix during the fermentation; the interactions of live cells with commensal microbiota in the gut environment; and the release and/or the induction of signalling molecules able to mediate complex physiological communications. However, probiotic effects of dairy LABs mostly can be mixed up with the potential health benefits of fermented milk products in terms of food–gut–brain axis perspective. Whether they are probiotic or starter culture for fermentation and also novel food additives for increasing functionality of products, they have positive health modulating effects on health in every way.

In recent years, the beneficial effects of dairy LAB consumption as probiotics and sources of bioactive metabolites have been extensively investigated with particular attention to their antimicrobial and immunomodulatory potential. However, recent evidence about their contribution to NCDs opens new therapeutic and prophylaxis perspectives that need further elucidations. Still, it is also obvious that there are many issues that need to be deeply revealed in terms of their potential for use (dose, duration, type, stability, etc.), especially in dietetic applications although there are many observational data in the literature. In particular, future investigations should be addressed to clarify the impact of dietary style on the gut microbiota composition; the underlying biochemical mechanisms of the probiotic supplementation by human clinical trials; and the impact of probiotic intake to induce long-term colonization of the gut microbiome.

Although dairy LAB may be a potential microbiome-targeted option in the treatment of NCDs, further studies are needed to identify specific strains and elucidate possible mechanisms to provide beneficial results in clinical trials.

## Figures and Tables

**Table 1:** Defined lactic acid bacteria (LAB) and their most common characteristics [20].

LAB	Family	Genus	Gram −/+	Growth Conditions			Type of Lactic Acid
				Heat-Stable (45 °C)	Salt-Tolerant (18% NaCl)	Acid-Resistant (pH 4.4)	
Dairy							
	*Lactobacillaceae*	*Lactobacillus*	+	Changeable	-	Changeable	D, L, DL
		*Pediococcus*	+	Changeable	-	+	L, DL
	*Streptococcaceae*	*Streptococcus*	+	Changeable	-	-	L
		*Lactococcus*	+	-	-	Changeable	L
	*Propionibacteriaceae*	*Propionibacterium*	+	-	-	-	
	*Enterococcaceae*	*Enterococcus*	+	+	-	+	L
	*Leuconostocaecae*	*Leuconostoc*	+	-	-	Changeable	D
Nondairy							
	*Aerococcaceae*	*Aerococcus*	+	-	-	-	L
	*Carnobacteriaceae*	*Carnobacterium*	+	-	-	NA	L
	*Enterococcaceae*	*Tetragenococcus*	+	-	+	Changeable	L
	*Enterococcaceae*	*Vagococcus*	+	-	-	NA	L
		*Fructobacillus*	+	NA	-	NA	D
	*Leuconostocaecae*	*Oenococcus*	+	-	-	Changeable	D
		*Weissella*	+	-	-	Changeable	D, L

NA: Not available, D: Dextrorotary; optical rotation to the right (+), L: Levorotary; optical rotation to the left (−).

**Table 2:** Some LABs isolated from dairy products, Their Potential Bioactivities & Probiotic Potentials and Stability Issues.

Dairy Products	Isolated Probiotic Strains	Their Bioactivities and Stability Issues	Reference(s)
Kalarei, a traditional fermented cheese product	*Pediococcus acidilactici* SMVDUDB2	* An 80% survival rate at low pH (2.0 and 3.0) and high bile salt concentration (0.3 and 0.5%) * High hydrophobicity affinity (33.3%) with ethyl acetate * Autoaggregation (77.68 ± 0.68%) and coaggregation (73.57 ± 0.47%) with *Staphylococcus aureus* (MTCC 3160) * Antibacterial activity against *Bacillus subtilis* (MTCC 121), *Mycobacterium smegmatis* (MTCC 994), *Staphylococcus aureus* (MTCC 3160), *Proteus vulgaris* (MTCC 426), *Escherichia coli* (MTCC 1652), and *Lactocaseibacillus rhamnosus* (MTCC 1408)	[37]
Ezine cheese (a Turkish cheese)	*Enterococcus lactis* PMD74	* The strain showed autoaggregative (41%) and coaggregative properties along with high viability at acidic pH (3.0) and in the presence of pepsin, pancreatin, and bile salts (0.3% and 0.5%). * The strain PMD74 inhibited the growth of a number of Gram-positive bacteria (*Listeria monocytogenes, Lactobacillus sake, Staphylococcus aureus*, and *Enterococcus faecalis*).	[36]
Tulum cheese (a Turkish cheese)	Seven *Limosilactobacillus fermentum* strains	* *Limosilactobacillus fermentum* LP3 and LP4 were able to tolerate acidic pH (2.5) and 1% bile salt. * Although all strains had similar enzymatic activity and antibiotic resistance patterns, the highest antagonistic effect belonged to LP3, LP4, and LP6 and the highest cholesterol assimilation belonged to LP3 and LP4, respectively.	[39]
Probiotic yogurt	*Lactobacillus acidophilus, Bifidobacterium bifidum, Lactiplantibacillus plantarum, Lacticaseibacillus casei*	* A combination of *Lactobacillus acidophilus* and *Bifidobacterium bifidum* survived at pH 1.5 during an incubation period of 1.5 h and also showed good survivability at 0.3% bile salt concentration. * At pH 2.0, 3.0, and 4.0, the survivability rate for *Lactobacillus acidophilus* and *Bifidobacterium bifidum* was 54, 66, and 64%, respectively.	[40]
Yogurt	*Streptococcus thermophilus* BGKMJ1-36 and *Lactobacillus bulgaricus* BGVLJ1-21	* Both strains grew at 37 and 45 °C in GM17 broth, while they did not grow in GM17 broth with 2% NaCl. * Both strains showed antimicrobial activity toward *Listeria monocytogenes,* while the BGKMJ1-36 strain produced EPS. * The colonies of BGKMJ1-36 and BGVLJ1-21 strains that successfully survived transit of the yogurt via simulated gastrointestinal tract conditions have been examined for adhesion to intestinal epithelial Caco-2 cells.	[41]
Iranian traditional yogurts	12 LAB isolates from two genera (*Pediococcus;* 6 *P. acidilacticii* isolates and *Lactobacillus*; 2 *Lactiplantibacillus plantarum*, 2 *Levilactobacillus brevis*, 1 *Limosilactobacillus fermentum* and 1 *Lactobacillus kefiri* isolates).	* *Limosilactobacillus fermentum* 27 had the highest acid tolerance, while *Levilactobacillus brevis* 25 had the highest bile salt tolerance. * *Pediococcus acidilactici* 23 showed a lower acid tolerance as well as *Levilactobacillus Brevis* 86 exhibited a lower bile salt tolerance than others.	[35]
Local dairy (cow milk, buffalo milk, cheese, and yogurt)	*Lactobacillus alimentarius, Lactobacillus sake,* and *Lactobacillus collinoides*	* The *Lactobacillus* strains inhibited pathogens’ growth. * All three isolates showed moderate activity apart from *Lactobacillus collinoides* and *Lactobacillus alimentarius,* which had relatively strong activity against *Pseudomonas aeruginosa* and *Bacillus subtilis*.	[42]
30 dairy samples (household milk and curd)	12 *Lactobacillus* isolates (LBS 1-LBS 12)	* Eight isolates (LBS 1-6, 8 and 11) were bile resistant (survival >50% at 0.3% bile salt *w*/*v*) and five isolates (LBS 1, 2, 5, 6 and 11) were resistant at acidic pH (survival >50% at pH 3). * All isolates inhibited the growth of Staphylococcus aureus. * LBS 2 also inhibited the growth of *Escherichia coli* and *Salmonella typhimurium*. * Isolate LBS 2 was resistant to five antibiotics as well as *Lactocaseibacillus rhamnosus* LBS2 successfully adhered to rat epithelial cells in in vitro conditions.	[43]
Traditional Greek dairy products (Feta, Kasseri, Xynotyri, Graviera, Formaela, Galotyri, and Kefalotyri cheeses as well as yogurt and milk)	25 LAB strains	* Only *Streptococcus thermophilus* ACA-DC 26 (Greek yogurt isolate) had antimicrobial activity (against *Streptococcus mutans* LMG 14558^T^). * Two *Lactiplantibacillus plantarum* strains (ACA-DC 2640 and ACA-DC 4039) showed the highest adhesion according to a collagen-based microplate assay and by using HΤ-29 and Caco-2 cells. * Milk cell-free supernatants of *Lactiplantibacillus* *plantarum* ACA-DC 2640 and ACA-DC 4039 showed strong angiotensin I-converting enzyme inhibition. * *Lactiplantibacillus plantarum* ACA-DC 2640, *Streptococcus thermophilus* ACA-DC 26, and ACA-DC 170 had anti-inflammatory activity.	[44]
Tibetan kefir	*Lactobacillus kefiranofaciens* XL10	* XL10 survived 3-h incubation at pH 3.5 and exhibited cell surface hydrophobicity of ~79.9% and autoaggregation of ~27.8%. * XL10 successfully adhered to the mucous tissue and colonized the ileum of the mice. * XL10 modulated gut microbiota by increasing the *Bifidobacteriaceae* family and decreasing in Proteobacteria phyla.	[45]
Mongolian fermented koumiss	*Lactobacillus helveticus* NS8	* Although NS8 exhibited a moderate survival ability in the gastrointestinal tract environment in vitro, an excellent adhesion ability to human intestinal cells and significant autoaggregation and cell-surface hydrophobicity were reported. * NS8 was able to decline the proinflammatory effects of lipopolysaccharide by inducing higher levels of IL-10.	[38]

LAB: Lactic acid bacteria, EPS: Exopolysaccharides.4. Gut–Brain Axis of Dairy LABs.

**Table 3:** Relationship between LAB isolated from dairy foods and non-communicable diseases (NDCs): A summary.

References	Health Effect	Study Design	Intervention	Intervention Duration	Dairy Product Type	Milk Heat Treatment	LAB Species	Main Results
ANIMAL MODELS, IN VITRO AND IN VIVO STUDIES								
[58]	Cardiometabolic markers and intestinal microbiota	8-weeks-old C57BL/6J wild-type (WT) and atherosclerotic (LRKO) male mice	HFD/high-sucrose diet [66% kcal lipids, 22% kcal carbohydrates (100% sucrose), 12% kcal proteins]. Protein sources groups: 1. 100% non-dairy protein (NDP) 2. 50% of the NDP energy replaced by milk protein (MP) 3. *Lactobacillus helveticus* fermented milk protein (FMP) 4. Greek-style yogurt protein (YP)	12 weeks (WT mice) 24 weeks (LRKO mice)	Yogurt and fermented milk	90 °C	- FMP: *Lactobacillus helveticus* - YP: *Streptococcus thermophilus* and *Lactobacillus delbrueckii* subsp. bulgaricus	- FMP and YP modulated the intestinal microbiota composition, upregulating the *Streptococcus* genus in both genotypes. - YP increased the expression of genes involved in jejunal and ileal immunity and integrity in WT mice; as well as, YP also improved insulin sensitivity by 65% in LRKO mice. - FMP attenuated hepatic inflammation, while both FMP and YP decreased circulating adhesion molecules. - Energy intake, body weight, fat mass, fasting glycemia, and insulinemia are unaffected by any of the dairy protein products.
[121]	Dextran sulfate sodium-induced colitis and intestinal microbiota	8-weeks-old specific-pathogen-free BALB/c female mice	1. Interventional colitis group (DSS-YC group) was administered 200 μL/d per mouse of the YC mixture (~5 × 10^9^ cfu) intragastrically. 2. The control colitis group (DSS group) received saline intragastrically as a vehicle. 3. Healthy control group received normal drinking water ad libitum (without DSS)	8 days	Yogurt culture (YC) bacteria	Not applied	- *Lactobacillus bulgaricus* 151 - *Streptococcus thermophilus* MK-10	- YC mixture reduced disease symptoms and inflammatory responsiveness of host to DSS. - *Lactobacillus bulgaricus* 151 and *S. thermophilus* MK-10 showed high anti-inflammatory and immunomodulatory potential. - YC mixture upregulated the colon length and modified intestinal microbiota by increasing the amount and diversity of mucosa-associated microbes and decreasing the concentration of putrefactive short-chain fatty acids in the faecal contents. - The strains have the potential to modulate the intestinal mucosal and systemic immune systems, causing IgA production and the stimulation of regulatory T cells
[122]	Immunostimulatory effects	- C3H/HeJ mice - Iterferon-γ knockout (KO) mice on a BALB/c - Myeloid differentiation factor 88 KO mice on a BALB/c - Mice spleen cell culture	EPS and bacteria isolated from 3 yogurt varieties fermented with different starter cultures administered to mice at doses of 100 μg/mouse (EPS) and 10^9^ cfu/mouse (bacteria) Yogurt types 1. *Lactobacillus delbrueckii ssp. bulgaricus* OLL1073R-1 and *Streptococcus thermophilus* OLS3059 (OLL1073R-1 yogurt) 2. *Lactobacillus delbrueckii ssp. bulgaricus* OLL1245 and *Streptococcus thermophilus* OLS3059 (yogurt A) 3. *Lactobacillus delbrueckii ssp.bulgaricus* OLL1256 and *Streptococcus thermophilus* OLS3295 (yogurt B).	3 weeks	Yogurt	90 °C	- Strains of *Lactobacillus delbrueckii* ssp. *bulgaricus* and *Streptococcus thermophilus*	- OLL1073R-1 was the highest EPS producer (154.6 mg/kg) along with being the only strain that induced the production of IFN-γ in vitro. - EPS or OLL1073R-1 yogurt increased natural killer cell activity and induced IFN-γ production in spleen cells, while other yogurt types had no effects. - That IFN-γ stimulated with EPS was completely blocked with both anti-IL-12 and anti-IL-18 antibodies in vitro. - OLL1073R-1 had more immunostimulatory effects than *Streptococcus thermophilus* OLS3059.
[123]	Colon cancer	BALB/c mice	1. DMH group: Mice treated with carcinogen 1–2 dimethylhydrazine (DMH). 2. Yogurt-DMH-yogurt group: Mice fed with yogurt for 10 days, injected with DMH, and then fed again cyclically with yogurt. 3. Yogurt group: Mice fed cyclically with yogurt from the eighth week until the sixth month. 4. Yogurt supernatant group: Mice-fed yogurt supernatant (LAB bacteria content < 1.0 × 10^2^ cfu/mL). 5. Milk group: Mice fed cyclically nonfat milk as control 6. Non-treatment control group: Mice not given any special treatment	6 months	Yogurt	Commercial yogurt process	- *Lactobacillus delbrueckii* subsp. *bulgaricus* and *Streptococcus thermophilus*	- Yogurt sustained enzymes activities similar to or lower than group-6, and the enzyme activity was also lower than milk or yogurt supernatant groups. - Group-2 had lower enzymes activities than the tumour control group. - Feeding yogurt decreased procarcinogenic enzyme levels in the large intestine contents of mice bearing colon tumours. - Yogurt starter bacteria interact with the large intestine of the mice and prevent colon cancer.
[124]	Hyperlipidemia and obesity	Swiss mice	1. Normal group 2. Low-concentration LCYBJ02 (LC-YBJ02-L)-10^8^ cfu/kg 3. Medium-concentration LC-YBJ02 (LC-YBJ02-M)-10^9^cfu/kg 4. High-concentration LC-YBJ02 (LC-YBJ02-H) group-10^10^ cfu/kg 5. HFD group	6 weeks	Yak yogurt- a Tibetan dairy product	Not reported	- *Lacticaseibacillus casei* YBJ02 (LC-YBJ02)	- The weight growth rate in all LC-YBJ02 groups was significantly higher than that in the normal group but was significantly lower than in the HFD group. - High concentrations of LC-YBJ02 can decrease the level of cholesterol, triglycerides, and LDL along with increasing the fecal cholesterol levels. - LC-YBJ02 (especially LC-YBJ02-H) reduced the expression levels of PPAR*γ,* C/EBPα, SREBP-1c, and FAS mRNA. - The mRNA expression levels of PPAR*γ,* C/EBPα, SREBP-1c, and FAS in the LC-YBJ02-H group were closest to those in the normal group.
[125]	Hypertension	Rabbit lung cultures	Whey proteins were fermented with 34 LAB and their ability to inhibit ACE activity was compared.	48 h	Whey proteins	37 °C	- 34 LAB strains	- All the fermentates displayed varying ACE inhibitory ability. - Seven fermentates showed strong ACE inhibitory abilities between 84.70 ± 0.67 (*Pediococcus acidilactici* SDL1414) and 52.40 ± 2.1% with IC_50_ values between 19.78 ± 1.73 and 2.13 ± 0.7 mg/mL. - Low molecular weight peptides of *Pediococcus acidilactici* SDL1414 showed the strongest ACEI activity. - *Pediococcus acidilactici* SDL1414 may be a potential starter culture in the dairy industry to develop antihypertensive functional foods.
[126]	Obesity, hyperlipidemia, and inflammation	6-weeks-old specific- pathogen-free C57BL/6J mice	1. Normal: Low fat diet 2. Model: HFD 3. HFD + LP-CQPC02: intragastrically 1 × 10^9^ cfu/kg body weight 4. HFD+ L-carnitine: intragastrically (200 mg/kg body weight) 5. HFD+ LDSB: intragastrically 1 × 10^9^ cfu/kg body weight	8 weeks	Yogurt culture bacteria	Not reported	- *Lactiplantibacillus plantarum* CQPC02 (LP-CQPC02) and *Lactobacillus delbruechii* subsp. *bulgaricus* (LDSB)	- LP-CQPC02 had a lower organ (liver, epididymal fat, and perirenal fat) index and lower AST, ALT, triglyceride, and total cholesterol and LDL-C levels but higher HDL-C level in the serum and liver. - Weight gain in the LP-CQPC02 group was lower than that in the L-carnitine or LDSB group. - LP-CQPC02-treated obese mice also had lower serum levels of the TNF-α, IFN-γ, IL-6, and IL-1 β, as well as higher levels of IL-4 and IL-10. - LP-CQPC02 up-regulated mRNA and protein expression of lipoprotein lipase, PPAR-α, CYP7A1, CPT1 while down-regulated PPAR-γ C/EBP-α. - The properties of LP-CQPC02 were better than LDSB, which is commonly used in the dairy industry.
[127]	Obesity and hyperlipidemia	6-weeks-old male Sprague-Dawley rats	1. Normal diet (ND) with oral saline administration 2. HFD with oral saline administration 3. HFD + *Limosilactobacillus fermentum* TSI-fermented yogurt 4. HFD + *Limosilactobacillus fermentum* S2-fermented yogurt 5. HFD + mixed TSI and S2 strains (1:1) fermented yogurt	8 weeks	Yogurt	85 °C	- *Limosilactobacillus fermentum* TSI and *Limosilactobacillus fermentum* S2	- All HFD groups showed significantly higher weight and fat, serum cholesterol, and abdominal adipose tissue levels. - TSI and S2 groups had lower triglyceride levels, smaller abdominal adipocytes, and higher serum HDL-C than the HFD group. - *Limosilactobacillus fermentum* TSI reduces abdominal fat and improves blood lipid metabolism in HFD-induced obese rats.
[128]	Breast cancer	6-weeks-old female BALB/c mice	1. Control group; received water 2. Milk group; given non-fat milk 3. FM group; given milk fermented by *Lacticaseibacillus casei* CRL 431 (2 ± 1 × 10^9^ cfu/mL)	50 days	Fermented milk	Sterilized	- *Lacticaseibacillus casei* CRL 431	- FM delayed tumour development, and about 50% of mice possessed tumours until day 50. - IL-6 and chemokine MCP-1 concentrations significantly reduced in the FM group after tumour detection, compared with the animals that received milk or did not receive any special feeding. - FM group had a significantly lower number of viable 4T1 breast cancer cells than groups that received milk or water. - FM inhibited the invasiveness of tumour cells in some mice and tumour cells were not present in their blood. - FM showed the highest survival rate, with 50% of mice remaining alive at the end of the experiment.
[129]	Breast cancer	7–8 weeks-old female BALB/c mice	1. Milk group (M)-given unfermented non-fat milk 2. Milk group (M)-given unfermented non-fat milk	36 days	Fermented milk	Sterilized	- *Lacticaseibacillus casei* CRL431	- Capecitabine’s toxicity on 4T1 cells was improved by the immune cells from mice that received PFM. - PFM declined capecitabine side effects in all the mouse models and reduced intestinal mucositis and mortality. - PFM administration to mice under chemotherapy maintained the anti-cancer/anti-metastasis effect of capecitabine with similar or decreased values of serum IL-10, TNF-α, and IL-6. - PFM reduced metastasis without side effects and improved the host’s immune response.
[130]	Breast cancer	7–8 weeks-old female BALB/c mice	1. Milk group (M)-given unfermented non-fat milk 2. Probiotic Fermented Milk (PFM) group, given milk fermented by *Lacticaseibacillus casei* CRL 431 (2 ± 1 × 10^9^ cfu/mL)	60 days	Fermented milk	Sterilized	- *Lacticaseibacillus casei* CRL431	- PFM administration reduced metastasis in the lungs and increased the survival of the animals. - PFM decreased pro-inflammatory cytokines and, locally in the lungs (metastatic organs), decreased F4/80+ cells, principally IL-10/F4/80+ cells. - PFM group had the highest percentages for CD4+ and CD4+ CD8+ cells. - While IL-10, TNF-α, IFN-γ and IL-6 concentrations decreased significantly in PFM group that increased in mice from milk group.
[131]	Breast cancer	BALB/c mice	1. Water (normal and tumor control groups) 2. Non-fat milk (milk group) 3. Milk fermented by *Lacticaseibacillus casei* CRL 431 (FM group; 2 ± 1 × 10^9^ cfu/mL)	28 days	Fermented milk	Sterilized	- *Lacticaseibacillus casei* CRL431	- FM delayed tumour development compared to the other groups. - IL-10/IL-6 ratio was maintained near to 1 in the mice from tumour control and milk groups, but that ratio decreased significantly in the FM group. - IgA+ cells were significantly increased in the mammary glands in the FM group. - Mice-administered milk increased significantly CD4+ cells after tumour injection, while the CD8+/CD4+ ratio was increased in the FM group (1.57) compared to the tumour control group (0.80) and milk group (0.52).
[132]	ACE inhibitory activity and hypertension	-ACE inhibitory activity-in vitro	1. Control yogurt: Lactobacillus delbrueckii ssp. bulgaricus Lb1466 and Streptococcus thermophilus St1342 (10^8^ cfu/mL) 2. Probiotic yogurt: control yogurt culture + Lactobacillus acidophilus L10, Lacticaseibacillus casei L26 and Bifidobacterium lactis B94 (10^8^ cfu/mL)	28 days	Yogurt	85 °C	*Lactobacillus delbrueckii* ssp. *bulgaricus* Lb1466, *Streptococcus thermophilus* St1342, *Lactobacillus acidophilus* L10, *Lacticaseibacillus* *casei* and *Bifidobacterium lactis* B94	- All probiotic yogurts showed appreciable ACE inhibitory activity during the initial stages of storage compared with the control yogurt. - The best ACE inhibition was observed during the first and third weeks of storage. - In total, 8 ACE inhibitory peptides were characterized originating from αs2-casein (1), κ-casein (2), and β-casein, of which two well-known ACE inhibiting peptides, namely Val–Pro–Pro (VPP) and Ile–Pro–Pro (IPP), were identified.
[72]	Obesity and hyperlipidemia	4-week-old male Sprague-Dawley (SD) rats	Normal diet (ND) HFD HFD plus FWB (HDFWB) FWB: 11% Whey protein concentrate (WPC) 80, 2% skim milk powder, 10.3% sugar, and culture	4 weeks	Whey beverage	70 °C	- *Lactiplantibacillus plantarum* DK211 and *Lactococcus lactis*	- The food intake in the HDWFB group was significantly lower than that of the HFD group. - There was a significant decrease in total cholesterol, LDL-C, and triglycerides in the HDFWB group compared with the HFD group, but there was no significant difference in serum HDL-C levels among the experimental groups. - Rats ingesting FWB (the HDFWB group) showed a significant decrease in blood glucose levels and plasma levels of insulin, leptin, and ghrelin compared to the HFD group.
[80]	Obesity and hyperlipidemia	4-week-old male C57BL/6 mice	Chow diet-saline group (Chow-Saline group) Chow diet-DH5 group (Chow-DH5 group), 2 × 10^8^ cfu of *Lactobacillus kefiri* DH5 60% high fat diet-saline group (HFD-Saline group) High-fat diet-DH4 group (HFD-DH4 group), 2 × 10^8^ cfu of *Leuconostoc mesenteroides* DH4 High-fat diet-DH5 group (HFD-DH5 group), 2 × 10^8^ cfu of *Lactobacillus kefiri* DH5. High-fat diet-DH7 group (HFD-DH7 group) 2 × 10^8^ cfu of *Lactobacillus kefiri* DH7	6 weeks	Kefir culture	25 °C	- *Lactobacillus kefiri* DH5 - *Leuconostoc mesenteroides* DH4	- *Lactobacillus kefiri* DH5 showed 100% survivability in gastrointestinal environments and reduced 51.6% of cholesterol levels. - HFD-DH5 group showed significant upregulation of PPARα, FABP4, and CPT1 in adipose tissues compared to the HFD-saline group, suggesting that *Lactobacillus kefiri* DH5 consumption promoted lipid oxidation in HFD-fed mice - *Lactobacillus kefiri* DH7 did not affect the body weight of HFD-fed mice.
[83]	Obesity	4-week-old male C57BL/6J mice and 3T3-L1 preadipocyte cells	Control group; HFD containing 5% microcrystalline cellulose (MCC) HFD containing 5% BG HFD containing 5% EPS HFD containing 8% kefir-grain residue obtained after EPS (Res) HFD containing 5% BG HFD containing 5% EPS HFD containing 8% kefir-grain residue obtained after EPS (Res) HFD containing 5% EPS HFD containing 8% kefir-grain residue obtained after EPS (Res) HFD containing 8% kefir-grain residue obtained after EPS (Res)	4 weeks	Kefir grain	30 °C	Kefir grain culture	- The highly pure EPS extracted from kefir grains showed antiobesity properties both in vitro and in vivo. - EPS suppressed adipogenesis in 3T3-L1 adipocytes. - EPS significantly reduced body weight gain, adipose tissue weight, and plasma VLDL-C compared with those of the control. - EPS supplementation significantly enhanced the abundance of the genus Akkermansia in feces. - BG, but not EPS, increased the abundance of the genus Allobaculum in faeces.
**HUMAN STUDIES (RCTs)**								
[77]	Obesity	210 Japanese adults with large visceral fat areas (80.2–187.8 cm^2^) 35–60 years	Fermented milk groups: Containing 10^7^ cfu LG2055/g, 200 g FM/d Containing 10^6^ cfu LG2055/g, 200 g FM/d Containing 0 cfu LG2055/g (control), 200 g FM/d	12 weeks	Fermented milk	40 °C	- *Lactobacillus gasseri* SBT2055 (LG2055) - *Streptococcus thermophilus* and *Lactobacillus delbrueckii* ssp. *bulgaricus*	- Abdominal visceral fat areas changed from baseline by an average of-8.5% (*p* < 0.01) in the 10^7^ dose group, and by-8.2% (*p* < 0.01) in the 10^6^ dose group. - BMI, waist and hip circumferences, and body fat mass were significantly decreased at the end of the experiment in both groups. - In the control group, none of these parameters significantly decreased from baseline.
[102]	T2DM	100 obese prediabetic males (BMI ≥ 25), (1-h post-load plasma glucose (PG) levels ≥180 mg/dL) ears	LcS-fermented milk contained >1.0 × 10^11^ cfu/100 mL Placebo: non-fermented milk with the same nutritional content, color, flavor, taste, and pH made using the same ingredients as the LcS-fermented milk	8 weeks	Fermented milk	Not reported	*Lacticaseibacillus casei* strain Shirota (LcS)	- In each group, body weight, BMI, and percentage of body fat significantly increased. - There were no statistically significant differences between the groups in terms of diastolic blood pressure. - HbA1c levels at 8 and 12 weeks were significantly reduced compared with baseline in the LcS group.

ACE: Angiotensin-converting enzyme; BMI: Body mass index; EPS: Exopolysaccharide; HDL-C: High-density lipoprotein cholesterol; HFD: High-fat diet, LDL-C: Low-density lipoprotein cholesterol; mRNA: Messenger ribonucleic acid; T2DM: Type 2 diabetes mellitus; VLDL-C: Very low-density lipoprotein cholesterol.
